# Comment on Huang et al. Colistin Monotherapy versus Colistin plus Meropenem Combination Therapy for the Treatment of Multidrug-Resistant *Acinetobacter baumannii* Infection: A Meta-Analysis. *J. Clin. Med*. 2022, *11*, 3239

**DOI:** 10.3390/jcm11237029

**Published:** 2022-11-28

**Authors:** Vered Daitch, Mical Paul, Leonard Leibovici

**Affiliations:** 1 Department of Medicine E, Rabin Medical Center, Beilinson Hospital, Petah-Tikva 4941492, Israel; 2 Sackler Faculty of Medicine, Tel Aviv University, Tel Aviv 6997801, Israel; 3 Infectious Diseases Institute, Rambam Health Care Campus, Haifa 3109601, Israel; 4 The Ruth and Bruce Rappaport Faculty of Medicine, Technion-Israel Institute of Technology, Haifa 3200003, Israel; 5 Research Authority, Rabin Medical Center, Beilinson Hospital, Petah-Tikva 4941492, Israel

In the recently published meta-analysis titled “Colistin Monotherapy versus Colistin plus Meropenem Combination Therapy for the Treatment of Multidrug-Resistant *Acinetobacter baumannii* Infection: A Meta-Analysis”, Huang et al. compared the efficacy and safety of treatment with colistin monotherapy versus colistin plus meropenem combination therapy in patients with drug-resistant *Acinetobacter baumannii* infection [1]. This comment addresses an error in the results of the published meta-analysis. The study included ten publications—three were reported as randomized controlled trials (RCTs) and seven as retrospective studies. All three “RCTs” were one trial including the same patients. The publication by Paul et al. described the results of the trial [2]. The other two studies were observational sub-studies: Dickstein et al. performed an exploratory subgroup analysis of the trial [3], and Nutman et al. tested the association between the presence of in vitro synergism and the trials’ clinical outcomes [4]. This is clearly reported in the secondary publications of the trial [3,4]. All three studies represent the same trial, but they were entered into the forest plots as if they were three different RCTs, repeatedly including the same patients, and creating a ‘unit-of-analysis error’, resulting in an exaggerated weight to the meta-analysis [5].

Another trial that should be mentioned is the OVERCOME trial by Kaye K. et al.: ‘Trial for the Treatment of Extensively Drug-Resistant Gram-negative Bacilli (OVERCOME)’. The trial results were not fully published yet but are available online on ClinicalTrials.gov [6].

In summary, the results of the meta-analysis should be corrected to include one report of the RCT by Paul et al. In addition, mentioning the OVERCOME trials’ results should be considered.
